# Vitamin D Reverts the Exosome-Mediated Transfer of Cancer Resistance to the mTOR Inhibitor Everolimus in Hepatocellular Carcinoma

**DOI:** 10.3389/fonc.2022.874091

**Published:** 2022-04-25

**Authors:** Mariarosaria Negri, Feliciana Amatrudo, Annalisa Gentile, Roberta Patalano, Tatiana Montò, Cristina de Angelis, Chiara Simeoli, Rosa Pirchio, Renata Simona Auriemma, Annamaria Colao, Rosario Pivonello, Claudia Pivonello

**Affiliations:** ^1^ Dipartimento di Medicina Clinica e Chirurgia, Sezione di Endocrinologia, Università Federico II di Napoli, Naples, Italy; ^2^ United Nations Educational, Scientific and Cultural Organization (UNESCO) Chair for Health Education and Sustainable Development, Federico II University, Naples, Italy

**Keywords:** exosomes, HCC, everolimus, drug resistance, PI3K/Akt/mTOR pathway

## Abstract

Several multi-kinase inhibitors were widely tested as potential first-line or second-line therapy in patients with advanced hepatocellular carcinoma (HCC). However, acquired drug resistance limits their clinical efficacy. Exosomes are microvesicles secreted by tumor and stromal cells that participate in many biological processes, including drug resistance. The current study evaluated the capability of exosomes derived from everolimus (EVE)-resistant HCC cells in inducing drug resistance in parental human HCC cells and the effect of 1,25(OH)_2_Vitamin D (VitD) treatment in restoring EVE sensitivity. The internalization of exosomes from EVE-resistant (EveR) cells into parental cells conferred the transmission of aggressive phenotype by promoting the transition of epithelial-to-mesenchymal phenotype, as demonstrated by immunofluorescence, and the acquisition of EVE resistance, as demonstrated by cell proliferation and colony formation assays. Moreover, the internalization of exosomes from EveR into parental cells induced deregulation of the mTOR pathway mainly by triggering the activation of the serine/threonine protein kinase Akt, involved in the cellular survival pathway, as demonstrated by Western blot analysis. Interestingly, the treatment with VitD prevented exosome-induced EVE resistance in HCC cells, significantly inhibiting cell proliferation but also partially reducing colony and size number when combined with EVE compared with control. In conclusion, the results of the current study demonstrated that exosomes derived from EveR cells could induce EVE resistance in EVE-sensitive HCC cells and that VitD can revert the exosome-induced EVE resistance by resensitizing to EVE treatment.

## Introduction

Hepatocellular carcinoma (HCC) is the most frequent among primary liver cancers and represents the fourth most common cause of cancer-related death worldwide (1). Cancer surveillance is applied to patients with a high risk to develop HCC, such as patients with cirrhosis, hepatitis B, and chronic hepatitis C with liver fibrosis, with the aim to detect tumors at early stages in order to increase the opportunity to use curative treatments and improve survival (1, 2). Nevertheless, regrettably, HCC is often diagnosed at advanced stages (1, 2). Currently, available or emerging systemic therapies, including multi-kinase inhibitors, used in advanced stages, still have limited efficacy with a scarce impact on overall survival (2). As observed in several types of cancers, systemic therapy often results in the reduction of tumor size but rarely succeeds in eradicating the totality of cancer cells, mainly for the acquisition of drug resistance, which is responsible for therapy failure (3). The phenotypic diversity of neoplastic cells that characterizes a tumor mass is considered a major driver of the development of resistance to medical therapy (3). Drug resistance development can involve several concomitant causes and mechanisms, including the drug efflux mechanisms, the persistence of cancer stem cells, the switch of the epithelial–mesenchymal transition (EMT), and the role of cancer-secreted microRNAs (miRNAs) and exosomes in the tumor microenvironment (4). Exosomes are the smallest extracellular vesicles with a diameter size ranging between 40 and 150 nm and a spherical shape bounded by a lipid bilayer membrane (5). Carrying a multitude of diverse biological factors, the exosomes can play a role in the different physiological and pathological processes (5). The different content of exosomes likely reflects the phenotypic states of the cells in physiological and pathological conditions. Interestingly, the exosomal cargoes can be conveyed to the neighboring or distant cells modulating the phenotype of these target cells (5) and, therefore, among the several effects, altering their capability to respond to the medical treatment. A previous study demonstrated that HCC cell-derived exosomes promoted resistance to the multikinase inhibitor sorafenib in *in vitro* and *in vivo* HCC models, and exosomes derived from highly invasive tumors could trigger stronger drug resistance (6). However, to the best of our knowledge, no studies have reported the ability of exosomes from HCC cell lines resistant to mTOR inhibitors in inducing drug resistance in HCC parental cells. Therefore, the primary aim of the current study was to investigate whether exosomes released by human everolimus (EVE)-resistant cell lines, JHH-6 and PLC/PRF/5, were able to induce EVE resistance in JHH-6 and PLC/PRF/5 parental cells. Moreover, the recent literature highlights the ability of 1,25(OH) vitamin D (VitD) to reverse the pharmacological resistance acquired in different tumor models and at different levels including the regulation of cancer stem cell growth (7), the EMT (7), and the regulation of specific miRNAs (7, 8). In particular, a recent work of the authors have demonstrated that a pre-treatment with VitD was able to restore the sensitivity to EVE in EVE-resistant HCC cell lines by regulating the EMT process and by reducing oncogene expression through the upregulation of miR-375 expression (8). Therefore, the secondary aim of the current study was to explore the role of VitD in the exosome-mediated transfer of cancer EVE resistance in HCC cell models.

## Materials and Methods

### Cell Cultures and Compounds

Human HCC cell lines PLC/PRF/5 and JHH-6 were used for the study. Parental cells were cultured as previously described (8). PLC/PRF/5- and JHH-6-resistant cells (EveR) were obtained by continuous culture of cell lines in the presence of EVE 10^-8^ M for at least 4 months (8). PLC/PRF/5 and JHH-6 parental cells were treated for 16 days with exosomes isolated from PLC/PRF/5 EveR and JHH-6 EveR cells and defined Exo EveR along with the text. Briefly, 1 × 10^6^ PLC/PRF/5 parental cells, 0.5 × 10^6^ JHH-6 parental cells, 2 × 10^6^ PLC/PRF/5 EveR cells, and 1.5 × 10^6^ JHH-6 EveR cells were seeded simultaneously in four different 75-cm^3^ flasks in the presence of cell culture exosome-free medium (fetal bovine serum, exosome-depleted, One Shot format, Gibco). Exactly every 3 days, the exosomes were extracted by EveR exosome-free medium by the Cell Culture Exosome Purification Midi Kit [Norgen Biotek Corp. (Canada)] according to the manufacturer’s instructions and inoculated in the flasks of both parental cells, respectively. When parental cells reached the confluence, they were detached by trypsin, split, and seeded again in order to complete the exosome-treatment cycle. After 16 days of exosome internalization, the cells were collected for the activity assays. Exosome-treatment protocol has been shown and detailed in **Supplementary Figure 1**. All cell lines were grown at 37°C in a humidified atmosphere with 5% CO_2_.

EVE [Selleck Chemicals (UK)] was dissolved in DMSO 100% and stored at −80°C. Fresh aliquots were defrosted prior to each new experiment. Serial dilutions were prepared using DMSO 40% reaching final concentrations of 0.04% in the medium in each well.

### Exosomal Protein Extraction and Western Blot

PLC/PRF/5 parental cells (1.5 × 10^6^), (1 × 10^6^) JHH-6 parental cells, (2 × 10^6^) PLC/PRF/5 EveR cells, and (1.5 × 10^6^) JHH-6 EveR cells were seeded in 75-cm^3^ flasks in the presence of cell culture exosome-free medium (fetal bovine serum, exosome-depleted, One Shot format, Gibco). Cells were grown in adhesion for 3 days, after which they reached 70% of confluence. Exo EveR cells were grown as described in the protocol detailed in **Supplementary Figure 1**. Parental, EveR, Exo EveR PLC/PRF/5, and JHH-6 cell pellets were harvested and lysed for protein extraction.

Moreover, proteins were also isolated from exosomes released in exosome-free medium of Exo EveR cells by using an ultracentrifuge according to the following protocol. The medium was centrifuged at 300 × *g* for 10 min at 4°C to remove cell debris. Subsequently, the medium was subjected to 3 other ultracentrifugations to eliminate, based on their size, the large vesicles (2,000 × *g* for 30 min at 4°C) and medium vesicles (16,500 × *g* for 30 min at 4°C) and obtain only the smaller vesicles (exosomes) (120,000 × *g* for 2 h at 4°C). The pellet, composed only of exosomes, is stored at −80°C until protein extraction.

Exosomal proteins were isolated in NP40 buffer supplemented with protease phosphatase inhibitor cocktail and an additional 2.0% SDS on ice for 30 min. The homogenate was centrifuged for 15 min at 1,200 × *g* and 4°C, and the supernatant was collected and stored at −80°C until use. Exosomal protein concentrations were determined photometrically with a bicinchoninic acid (BCA) Protein Assay Kit (Thermo Scientific, USA).

After protein heat denaturation at 95°C for 10 min, 5 µg of total extracts was used for immunoblotting. Exosomal proteins were separated by 8% SDS-PAGE and then electroblotted onto a nitrocellulose membrane for 90 min in a TransBlot apparatus. After a blocking treatment for 1 h with 5% milk, the nitrocellulose filters were probed with primary antibodies specific for ALIX (EPR15314-33, Abcam), TSG101 [EPR7130(B), Abcam], and CD9 (EPR2949, Abcam) overnight. Subsequently, filters were hybridized with peroxidise-conjugated secondary antibodies and immunoreactive bands were detected by the ECL system. After a chemiluminescent reaction, the blot was exposed to ImageQuant Las 4000 (GE Healthcare).

### Exosomes Isolation and Staining

PLC/PRF/5 parental cells (1.5 × 10^6^), (1 × 10^6^) JHH-6 parental cells, (2 × 10^6^) PLC/PRF/5 EveR cells, and (1.5 × 10^6^) JHH-6 EveR cells were seeded in 75-cm^3^ flasks and grown in 10 ml of exosome-free medium. After 3 days, the cells reached 70% confluence. The medium of EveR cells was collected and exosomes were isolated from cell culture exosome-free medium by using the Cell Culture Exosome Purification Midi Kit [Norgen Biotek Corp. (Canada)] according to the manufacturer’s instructions. Ten milliliters of cell culture media was transferred in a conical tube and centrifuged at 1,000 RPM for 15 min to remove any cells and debris. The cell-free media was transferred into a new 15-ml conical tube where 2.5 µl of ExoC Buffer was added for every 1 ml of cell-free media and 400 µl of Slurry E resin was then supplemented. The solution was mixed well by vortexing for 10 s, left at room temperature for 10 min, and then centrifuged for 2 min at 2,000 RPM. The supernatant was discarded and 400 µl of ExoR Buffer was applied to the slurry pellet; it was mixed well by vortexing for 10 s and the resuspended slurry pellet was incubated at room temperature for 10 min. After incubation, the resuspended pellet was vortexed for 10 s and centrifuged for 2 min at 500 RPM. Lastly, the supernatant was transferred to a Mini Filter Spin column assembled with an elution tube and was centrifuged for 1 min at 6,000 RPM. The flowthrough contained the purified exosomes.

Subsequently, in order to resuspend the exosomes in PBS 1X, the Exo-spin Exosome Purification Kit [Cell Guidance Systems (UK)] was used. Extracted exosomes (200 µl) were added to the column and it was centrifuged for 1 min at 50 × *g*; the eluate was discarded. The column was placed in a new Eppendorf and 200 µl of PBS 1X was added to the column and centrifuged at 50 × *g* for 60 s. The exosomes were then marked with PKH67 Green Fluorescent membrane staining [Sigma-Aldrich (USA)]. PKH67 dye (1 µl) was diluted in 500 µl of diluent C. Final dye (250 µl) was mixed with 200 µl of exosomes in PBS 1X for 4 min.

The reaction was stopped by adding an equal volume of PBS 1X and FBS exosome-free (1:1) to this mix, for 3 min. To remove the excess dye, the Exo-spin Exosome Purification Kit [Cell Guidance Systems (UK)] was used as previously described and the colored exosomes were contained in the eluate.

The stained exosomes were inoculated in µ-Dish 35 mm (IBIDI, Germany), previously prepared with PLC/PRF/5 and JHH-6 parental cells at 70% confluence to evaluate the exosome uptake after about 18 h. Images were visualized on an inverted microscope, Olympus IX51, equipped for fluorescence and phase-contrast microscopy (Olympus, Milan, Italy) and were captured at 40× magnification.

### mRNA Isolation and RT-qPCR

mRNA isolation from parental and resistant cells was carried out as previously described (9). mRNA expression pattern of single components of mTOR pathway, including mTOR, p70S6k, and 4eBP1, was assessed by SYBR Green-based RT-qPCR performed with specific sets of primer sequences as previously reported (10). The final product was subjected to graded temperature-dependent dissociation to verify that only one product was amplified. Reactions were run in duplicate, and each reaction was repeated thrice on a StepOne Plus real-time PCR machine (Applied Biosystems Foster City, CA, USA). The relative expression levels of each transcript analyzed in each sample were normalized using the housekeeping gene β-actin.

### Cell Proliferation Assay

After trypsinization, PLC/PRF/5 parental, EveR and Exo EveR cells (1.5 × 10^4^) and JHH-6 parental, EveR and Exo EveR cells (1.5 × 10^3^) were plated in 1 ml of complete culture medium in 24 well plates for 6 days, respectively. The plates were then placed in an incubator in 5% CO_2_ at 37°C. After 24 h, VitD 10^-7^ M and EVE were added to each well at different concentrations, ranging between 10^-11^ and 10^-7^ M. Controls were vehicle-treated. Plates were further incubated at 37°C and 5% CO_2_. The medium was changed and VitD and EVE were freshly added every 3 days. After 6 days of treatment, cells were harvested for DNA measurement. Measurement of total DNA content, representative for the number of cells, was performed using the bisbenzimide fluorescent dye (Hoechst 33258) (Boehring Diagnostics, La Jolla, CA), as previously described (11).

### Immunofluorescence Staining

Immunofluorescence staining was performed as previously described (8, 10). The cells were incubated with primary antibodies against Vimentin (Abcam, ab92547, rabbit monoclonal, dilution 1/250) and E-cadherin (Santa Cruz, sc-21791, mouse monoclonal, dilution 1/100) for 1.5 h. Slides were then washed thrice in 0.1% Triton/PBS for 5 min and incubated with the secondary antibodies for 1 h (Millipore, AP124F, goat anti-mouse, FITC conjugated, dilution 1/500; ImmunoReagent, Gtx-Rb-003-DRHO, goat anti-rabbit, TRITC conjugated, dilution 1/500). Nuclei were stained with 4,6-diamidino-2-phenylindole (DAPI) (Lonza Group Ltd, Basel, Switzerland), diluted in PBS 1X 1:40,000. Images were visualized on an inverted microscope, Olympus IX51, equipped for fluorescence and phase-contrast microscopy (Olympus, Milan, Italy) and were captured at 40× magnification and acquired with Olympus IX2-LWUCD 6A14956 Digital Camera F-View II (Olympus, Milan, Italy).

### Colony-Forming Assay

Colony-forming assay was performed as previously described (8). Parental PLC/PRF/5, EveR, and Exo EveR cells (500 cell/ml) were plated into six-well culture dishes and cultured in complete medium for 21 days. After 24 h of adhesion, cells were treated with EVE 10^-10^ M and/or VitD 10^-7^ M, whereas in control cells, vehicles were added. Every 3 days, a fresh medium was replaced and drugs and vehicles were readded. After 21 days, formed colonies were stained (12) and counted. Moreover, the dimension of colonies was evaluated by ImageJ software as integrated density.

### Cell Lysis and Western Blot

Cell lysis and WB analysis were performed as previously described (9). Primary antibodies specific for Akt (#9272, Cell Signaling Technology, Italy), p70S6k (H-9) (sc-8418) (Santa Cruz Biotechnology Inc.), 4eBP1 (53H11) (#9644, Cell Signaling Technology, Italy), pAkt (ser473) (#9271, Cell Signaling Technology, Italy), pp70S6k (Thr389) (#9206, Cell Signaling Technology, Italy), p4eBP1 (Ser65) (#9456, Cell Signaling Technology, Italy), and β-actin (A4700; Sigma-Aldrich, Italy) were probed on nitrocellulose filters overnight. Peroxidase-conjugated secondary antibodies (Donkey anti-Rabbit IgG, DkxRb-003-DHRPX, ImmunoReagents, Inc) (Goat anti-Mouse IgG, GtxMu-003-DHRPX, ImmunoReagents, Inc) used were probed for 1 h. Immunoreactive bands were detected by the ECL system and the blot was exposed to ImageQuant Las 4000 (GE Healthcare).

### Statistical Analysis

All the experiments were performed in quadruplicate and were replicated three times with the exception of Western blot analysis that was replicated two times. All statistical analyses were performed using GraphPad software. Based on the number of groups to compare, differences between controls and treated groups were determined by Student’s *t*-test or ANOVA. *p*-values less than 0.05 were considered statistically significant.

## Results

### Evaluation of Exosomes’ Extraction From Cell Media

**Figure 1:**
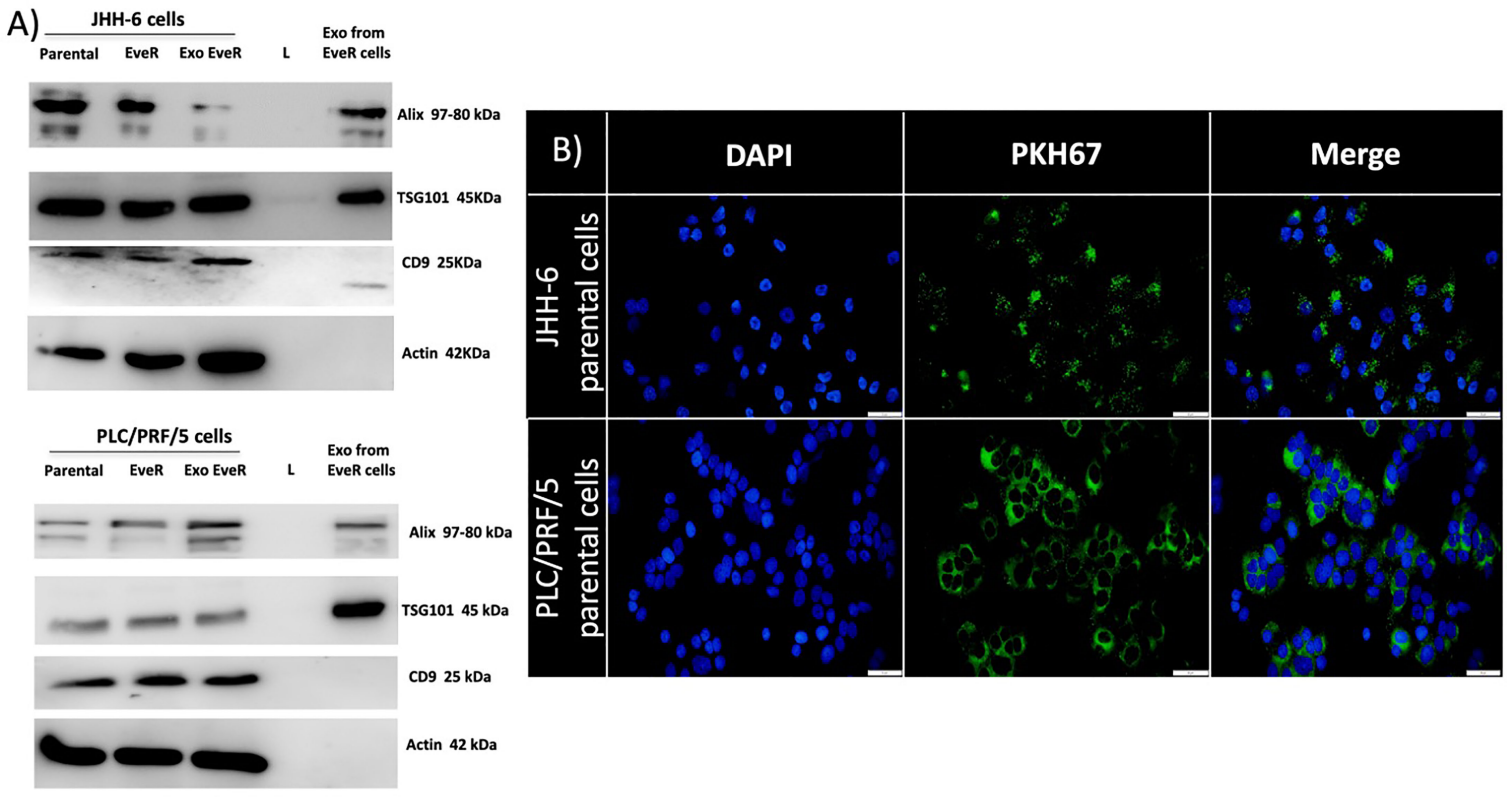
**(A)** Immunoblot analysis of ALIX, TSG101, CD9, and β-actin in whole JHH-6 and PLC/PRF/5 parental cells, EveR, and Exo EveR and in exosome from EveR cell lysates. L, ladder. **(B)** Green fluorescence microscopy showing the uptake of PKH67-labeled exosomes from JHH-6 and PLC/PRF/5 EveR cells to recipient JHH-6 and PLC/PRF/5 parental cells. Blue represents DAPI staining of the nuclei; green represents exosome membrane staining.

To confirm that the vesicular structures isolated by JHH-6 and PLC/PRF/5 EveR cell media are exosomes, proteins from the plasma and endosomal membranes, including programmed cell death 6-interacting protein (ALIX), tumor susceptibility gene 101 (TSG101), and cluster of differentiation 9 (CD9), were used as markers. As shown in **Figure 1A**, the specific and selective markers ALIX and TSG101 are both expressed in the exosomes released by JHH-6 and PLC/PRF/5 EveR, while CD9 is only expressed in the exosomes released by JHH-6 EveR. In the exosomal samples of both cell lines, no expression of the cytoskeleton marker, β-actin, confirmed the purity of the exosomal samples.

### Exosomes From HCC EveR Cells Internalize in HCC Parental Cells

To explore whether the exosomes from donor JHH-6 and PLC/PRF/5 EveR cells could be transferred to recipient JHH-6 and PLC/PRF/5 parental cells, the uptake of labeled exosome was investigated. As shown in **Figure 1B**, JHH-6 and PLC/PRF/5 EveR cell-derived exosomes, labeled with PKH67 Green Fluorescent Cell Linker, were inoculated with JHH-6 and PLC/PRF/5 parental cells and green staining in the parental cells was observed within 18 h, confirming the exosome incorporation.

### Exosomes Induce Mesenchymal-Like Markers in Parental Cells

**Figure 2:**
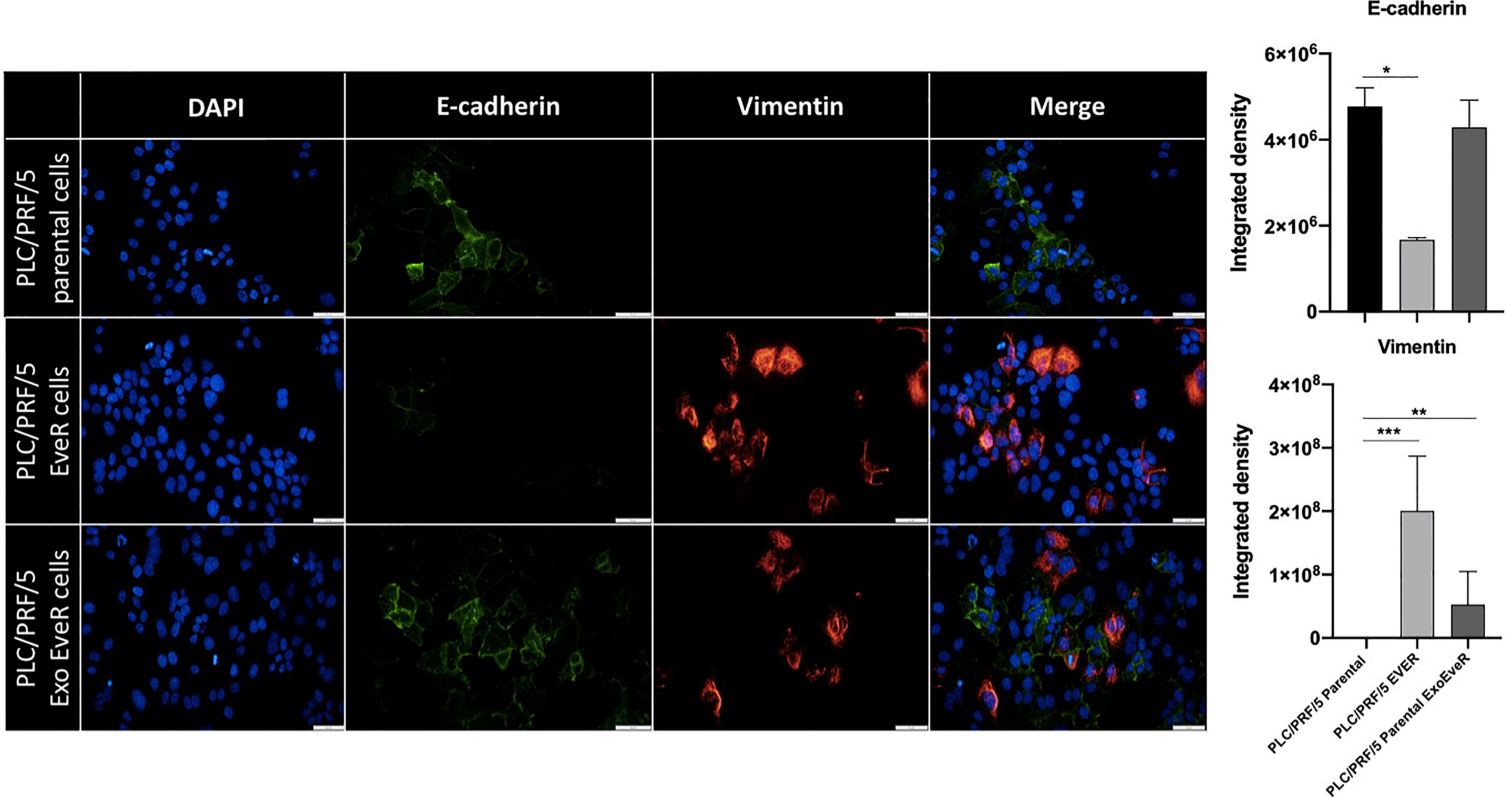
EMT markers in PLC/PRF/5 parental, EveR, and Exo EveR cells. Exosomes from PLC/PRF/5 EveR cells induce EMT in PLC/PRF/5 parental cells. Blue represents DAPI staining of nuclei; green represents FITC staining of E-cadherin protein; red represents TRITC staining of vimentin protein. *p < 0.05; **p < 0.01; ***p < 0.001.

Tumor progression and the development of drug resistance are dependent on several molecular factors including the crosstalk between tumor cells and their microenvironment. Exosomes released by tumor cells represent a means of communication between cells; therefore, they might take an active role in the transmission of drug resistance. To further investigate the effect of exosome cross-talk on spreading drug resistance signals, it has been tested whether, in parental cells, the incorporation of exosomes released by EveR cells could induce EMT that has been demonstrated to confer resistance to many types of therapeutic agents on tumor cells. Considering the well-known mesenchymal phenotype of parental JHH-6 (13, 14), the EMT has been investigated only the PLC/PRF/5 cell line. As shown in **Figure 2**, PLC/PRF/5 parental cells and PLC/PRF/5 EveR cells mainly express E-cadherin and vimentin, respectively. Interestingly, when exosomes released by PLC/PRF/5 EveR cells were incorporated by the recipient PLC/PRF/5 parental cells in the latter, a significant gain of EMT marker vimentin (*p* < 0.01) was observed.

### Exosome Internalization Induces Drug Resistance in HCC Parental Cells

**Figure 3:**
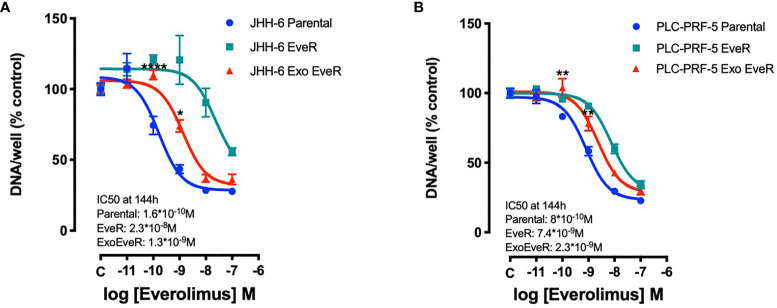
Exosomes are responsible for acquired EVE resistance in HCC cell lines. The graphs represent the proliferation of parental, EveR, and Exo EveR cells in JHH-6 **(A)** and PLC/PRF/5 **(B)** cell lines. Parental cells were exposed for 16 days to exosomes isolated by EveR cells (red lines) and then treated for 6 days at escalating doses of EVE. *p < 0.05, **p < 0.01, and ****p < 0.0001 compared to the effect reached after the same EVE treatment doses in parental cells.

To investigate whether EveR-derived exosome internalization has a role in the acquisition of resistance to EVE in parental cells, cell proliferation assay was performed in JHH-6 and PLC/PRF/5 parental, EveR, and Exo EveR cells after the exposure to escalating doses of EVE, from 10^-11^ M to 10^-7^ M, for 6 days. As shown in **Figures 3A, B** (green line), both HCC cell lines, continuously cultured for at least 4 months in the presence of EVE 10^-8^ M, acquired drug resistance to the cytostatic effect of escalating doses of EVE, confirming our previous results (8).

Additionally, as shown in **Figures 3A, B** (red line), following the internalization of exosomes, both JHH-6 and PLC/PRF/5 Exo EveR showed acquired resistance to EVE treatment as demonstrated by the significantly reduced efficacy in inhibiting the cell proliferation observed at the lower concentrations of EVE (10^-10^ M and 10^-9^ M) tested. Indeed, in JHH-6 parental cells, the EVE 10^-10^ M treatment induced 24.7% of inhibition compared to control, whereas the same treatment no longer induced any inhibition in JHH-6 Exo EveR (*p* < 0.0001 vs. EVE 10^-10^ M in JHH-6 parental cells). Accordingly, even the EVE 10^-9^ M treatment induced 56.5% of inhibition (*p* < 0.0001) in JHH-6 parental cells compared to control, whereas the same treatment in JHH-6 Exo EveR induced only 26.1% of inhibition compared to control, with a reduced percentage of inhibition (30.4%, *p* < 0.05) compared to the effect of the same EVE treatment in JHH-6 parental (**Figure 3A**). Consistently, in PLC/PRF/5 parental cells, the EVE 10^-10^ M treatment induced 16.8% of inhibition (*p* < 0.05) compared to control, whereas the same treatment no longer induced any inhibition in PLC/PRF/5 Exo EveR (*p* < 0.01 vs. EVE 10^-10^ M in PLC/PRF/5 parental cells). Accordingly, even the EVE 10^-9^ M treatment induced 41.7% of inhibition (*p* < 0.0001) in PLC/PRF/5 parental cells compared to control, whereas the same treatment in PLC/PRF/5 Exo EveR induced only 21.8% (*p* < 0.001) of inhibition compared to control, with a reduced percentage of inhibition (19.94%, *p* < 0.01) compared to the effect of the same EVE treatment in PLC/PRF/5 parental (**Figure 3B**).

The percentage of cell proliferation inhibition induced by EVE in PLC/PRF/5 and JHH-6 Exo EveR cells is summarized in **Table 1**.

**Table 1:** PLC/PRF/5 and JHH-6 Exo EveR cells acquired resistance to EVE as demonstrated by a reduced percentage of cell proliferation inhibition.

Cell lines	EVE 10-^10^ M		EVE 10-^9^ M	
	% of inhibition vs. C	*p*	% of inhibition vs. C	*p*
**Parental JHH-6**	24.6	< 0.0001	56.5	< 0.0001
**Exo EveR JHH-6**	i.n.i.	n.s.	26.1	n.s.
**Parental PLC/PRF/5**	16.8	< 0.05	41.7	< 0.0001
**Exo EveR PLC/PRF/5**	i.n.i.	n.s.	21.8	< 0.001

Parental and Exo EveR cells were exposed to serial EVE concentrations for 6 days. While parental cells displayed an evident dose–response curve (blue lines in **Figures 3A, B**) even at the lowest EVE concentrations (10^-10^ M and 10^-9^ M), Exo EveR cells showed loss of response to drug treatment at the lowest concentrations. i.n.i., inhibition not induced; n.s., not significant.

**Figure 4:**
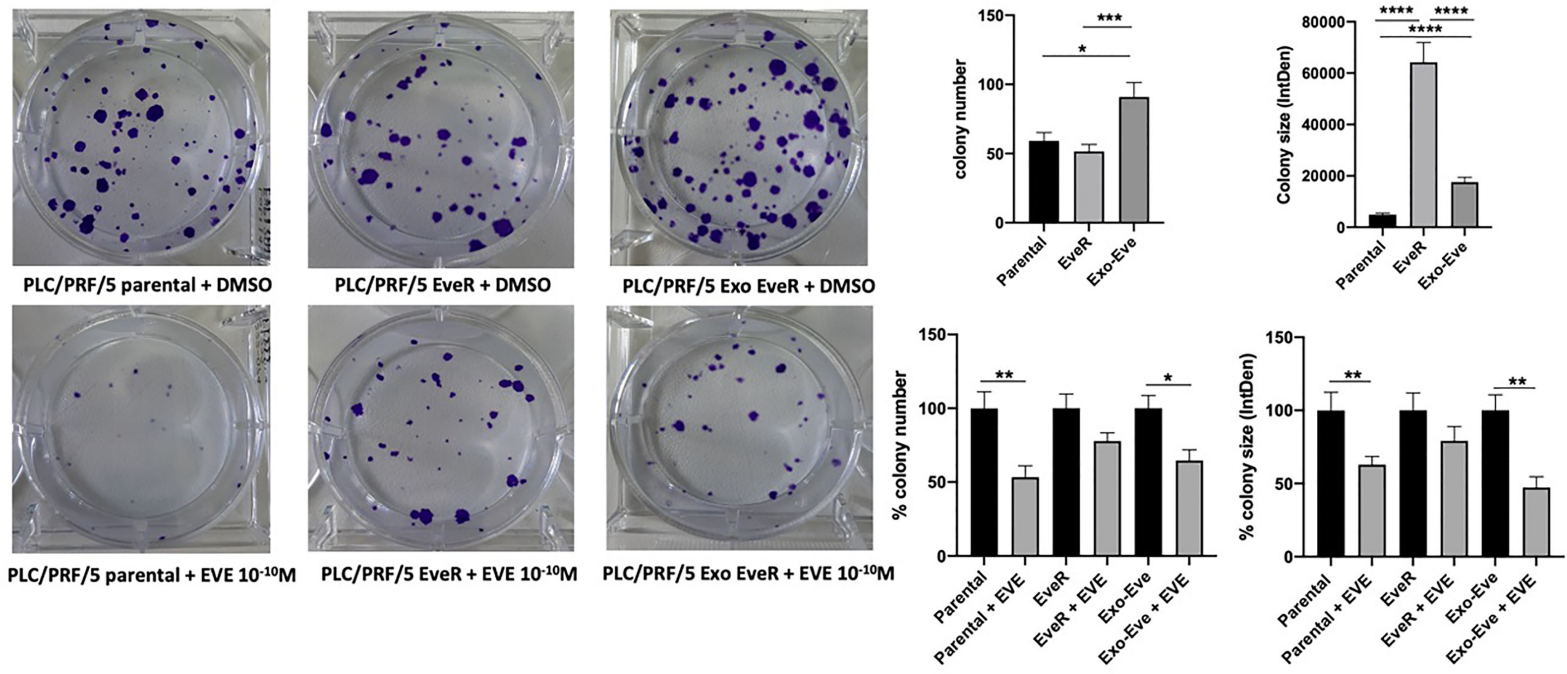
Exosomes are responsible for acquired EVE resistance in HCC cell lines. The images and graphs represent the colony size of parental, EveR, and Exo EveR cells in PLC/PRF/5 before and after EVE treatment. Parental cells were exposed for 16 days to exosomes isolated by EveR cells. All cells were treated for 10 days at EVE 10^-10^ M. *p < 0.05; **p < 0.01; ***p < 0.001; ****p < 0.0001.

As shown in **Figure 4**, a colony formation assay, performed with the PLC/PRF/5 cells that in contrast to JHH-6 cells were able to form colonies, demonstrated that the internalization of EveR-derived exosome in parental cells (PLC/PRF/5 Exo EveR) induced the formation of a higher colony number (*p* = 0.034) and higher colony size (*p* < 0.0001) compared to parental cells, suggesting that the internalization of EveR-derived exosome might induce a more aggressive phenotype. Nevertheless, the internalization of EveR-derived exosome does not induce EVE resistance compared to parental cells as observed in colony number formation. Indeed, EVE treatment in PLC/PRF/5 parental cells induces 46.7% (*p* = 0.006) of colony number inhibition and 37.0% (*p* = 0.009) of colony size reduction compared to the same untreated cells whereas EVE treatment in Exo EveR cells induces 35.3% (*p* = 0.012) of colony number inhibition and 52.7% (*p* = 0.001) of colony size reduction compared to the same untreated cells, with a non-significant difference between the effect observed in parental cells treated with EVE and that in Exo-EveR treated with EVE.

**Figure 5:**
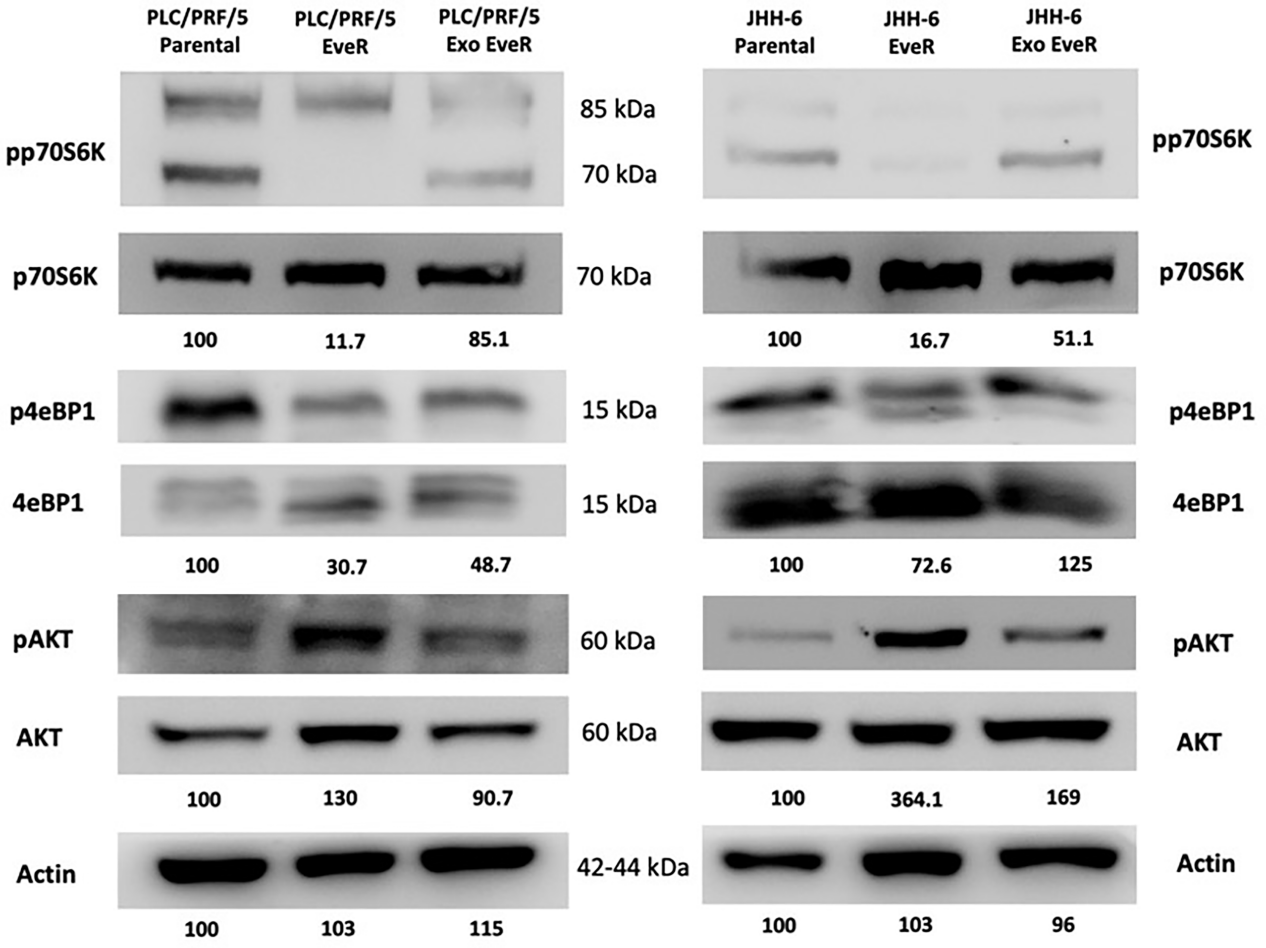
Immunoblot analysis of mTOR components in JHH-6 and PLC/PRF/5 parental, EveR, and Exo EveR cells. Densitometry analysis values represent the ratio of phosphorylated/total proteins and of actin as mean of two independent experiments.

As shown in **Figure 5**, according to the immunoblot analysis, the protein content of the different mTOR pathway components reflects the acquired resistance to EVE in JHH-6 and PLC/PRF/5 EveR cells. Indeed, a strong reduction of the activity of mTOR downstream effectors p70S6K and 4eBP1 was detected in both cell lines. Interestingly, the internalization of exosomes from EveR cells into parental cells induces a reduction in protein content of phosphorylated forms of p70S6k in both JHH-6 and PLC/PRF/5 Exo EveR cells and of 4eBP1 in PLC/PRF/5 Exo EveR cells, and concomitantly, a hyperexpression of the phosphorylated form of Akt was observed in JHH-6 Exo EveR cells compared to the parental cells.

### Vitamin D Reduces the Exosome-Mediated Transfer of Cancer Resistance to EVE in HCC Cell Lines

To investigate whether EveR-derived exosome internalization might be reduced by VitD, a cell proliferation assay was performed in both JHH-6 and PLC/PRF/5 Exo EveR, while a colony formation assay was performed only in PLC/PRF/5 Exo EveR, after the exposure to VitD 10^-7^ M, alone and in combination with EVE 10^-10^ M for 6 days, in the proliferation assay, and for 21 days, in the colony formation assay.

**Figure 6:**
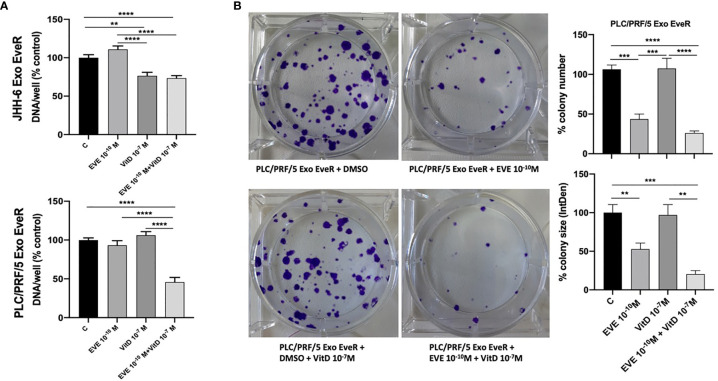
VitD reduces the exosome-mediated transfer of cancer resistance to EVE in the PLC/PRF/5 cell line. **(A)** The graph represents the proliferation of JHH-6 and PLC/PRF/5 Exo EveR cells after 6 days of treatment with EVE 10^-10^ M, VitD 10^-7^ M, and EVE 10^-10^ M + VitD 10^-7^ M. **p < 0.01; ****p < 0.0001. **(B)** The images and graph represent the colony number and size of PLC/PRF/5 Exo EveR treated for 21 days with EVE 10^-10^ M, VitD 10^-7^ M, and EVE 10^-10^ M + VitD 10^-7^ M. **p < 0.01; ***p < 0.001; ****p < 0.0001.

As shown in **Figure 6A**, VitD induces 23.4% (*p* < 0.01) of cell proliferation inhibition in JHH-6 Exo EveR, and no further significant inhibition was observed when VitD was combined with EVE compared to control, whereas VitD resensitizes the PLC/PRF/5 Exo EveR to the inhibitory effect of EVE, with 63% (*p* < 0.0001) of inhibition compared to control and with 50% (*p* < 0.0001) of inhibition compared to EVE alone.

Moreover, as shown in **Figure 6B**, when VitD was combined with EVE, a trend of further, although not significant, inhibition in colony number and size was also observed in PLC/PRF/5 Exo EveR compared to the effect induced by the treatment with the EVE alone. Indeed, EVE induced 63% (*p* < 0.001) while the combined treatment induced 80% (*p* < 0.0001) of inhibition of colony number compared to control. Accordingly, EVE induced 47% (*p* < 0.01) while the combined treatment induced 79% (*p* < 0.001) of inhibition of colony size compared to control.

## Discussion

The increased number of deaths due to HCC remains a growing concern, although in recent years, great progress has been achieved in diagnosis and therapeutic strategies for the clinical management of HCC (1, 2). Among systemic target therapies recommended for patients who have advanced disease, the multi-kinase inhibitors sorafenib, approved by the Food and Drug Administration (FDA) as a first-line treatment, and regorafenib, approved by FDA as a second-line treatment, have been demonstrated to be safe and effective in increasing survival rate (2). Several additional agents tested for second-line treatment, including the mTOR inhibitor EVE, did not increase the survival rate of advanced HCC (2). The response of a tumor to multi-kinase inhibitors or chemotherapy may be strongly influenced by microenvironmental factors (15). Indeed, solid tumors, including HCC, represent heterogeneous structures composed of cancer, stromal, and immune cells, surrounded by extracellular matrix, and sustained by aberrant vasculature, and exosomes secreted by cancer cells and released in the microenvironment can have an impact on oncogenesis, tumor progression, and drug resistance (15). Increasing evidence demonstrated that exosomes can directly transmit drug-resistant signals by mediating cargo signals including proteins, nucleic acids, and micro-RNA (miRNAs) and affecting EMT or cancer stem cell properties, and by influencing immune response (16–18). It is well known that exosomes have a role in the interactions between HCC tumor cells and their surrounding hepatic milieu. Indeed, a large number of pro-tumorigenic RNAs and proteins, such as MET proto-oncogene, S100 family members, and the caveolins, carried by exosomes from metastatic cell lines, can enhance the migratory and invasive abilities of non-motile immortalized hepatocyte cell line, by activating the mTOR and MAPK pathway (19). In the context of cancer, consisting of heterogeneous cell populations, sensitive cancer cells represent the main cell population that can be affected by multi-kinase inhibitor treatment. Using melanoma cell models, it has been proven that drug-sensitive cells can release “secretomes” driving and fostering the outgrowth of drug-resistant cells (20), and exosomes might represent the major part of these “secretomes” as demonstrated in *in vitro* and *in vivo* HCC models where cell-derived exosomes promoted resistance to sorafenib (6). Nevertheless, to the best of our knowledge, it is not clearly demonstrated in HCC models whether the drug-resistant cells can promote and sustain drug resistance in sensitive cancer cells, triggering their cell proliferation progression.

The primary findings of the current study support the hypothesis that exosomes derived from drug-resistant cells mediate tumor cell–cell communications promoting the resistance to EVE and, consequently, inducing tumor progression by the activation of cell proliferation and survival of sensitive HCC cancer cells. Exosomes derived from EVE-resistant cells bestow the mesenchymal phenotype and deregulate the mTOR pathway to sensitive cells, conferring the transmission of an aggressive phenotype. Accordingly, previous studies reported that exosomes derived from highly metastatic HCC cell lines can be taken up by lowly metastatic HCC cell lines undergoing EMT, showing a higher expression of mesenchymal markers and a lower expression of epithelial markers (21, 22). Moreover, previous findings reported by the authors demonstrated that the chronic exposure of HCC cell lines to EVE induces a change in cell phenotype, allowing the cells to acquire more aggressive features as confirmed by the higher expression of vimentin (8). In addition, the results of the current study prove that the “information” of acquired aggressiveness following chronic EVE exposure can be conveyed to sensitive cells through paracrine or endocrine cargo signals by exosomes in HCC *in vitro* models.

Moreover, the present findings reveal that the chronic EVE exposure induces the inhibition of phosphorylation in Thr389 and in Ser65 in both mTOR pathway components, p70S6K and 4eBP1, respectively, and the activation of Akt in Ser473 in both JHH-6 and PLC/PRF/5 cell models. These data are in line with what has been previously reported even in the BON-1 cell line, a model of pancreatic neuroendocrine tumor, in which a long-lasting (8 weeks) exposure to EVE induced mTOR pathway inactivation of pp70S6K on Thr389 and of p4eBP1 on Thr70 (23). Remarkably, the reduction of molecular target activity is one of best-known mechanisms of resistance to target-based agents. Dowling et al. reported that the key proteins of mTOR pathway p70S6K and 4eBP1 regulate cell growth and mediate cell proliferation, respectively, and notably, when 4eBP1 was knocked down, the anti-proliferative effect of EVE was decreased (24). Interestingly, depending on the cell models and solid tumors, EVE can inhibit the phosphorylation of 4eBP1 on several threonine and serine residues, including Thr70 and Ser65 (25–27), and consequently, the reduced 4eBP1 phosphorylation results in the attenuated effect of EVE that chronically induces EVE resistance. On the other hand, phosphorylation of Akt on Ser473 through the mTOR complex 2 tended to be stronger in EVE-treated cells of solid and hematological tumors, causing the insufficient anti-tumor effect of EVE and triggering the resistant properties of these cells (27–29). Accordingly, the results of the current study showed reduced phosphorylation of p70S6K in JHH-6 and PLC/PRF/5 Exo EveR and of 4eBP1 in PLC/PRF/5 Exo EveR, demonstrating that internalization of EveR exosomes transfers cargo signals capable of deregulating the activity of mTOR target molecules and inducing EVE resistance.

The role of VitD as a reversal agent of drug resistance is gaining growing interest in the scientific community as witnessed by the evidence accumulated during these recent years. Indeed, in several cancer models, VitD has been reported to sensitize tumor drug-resistant cells to chemotherapy and multi-kinase drugs acting through several mechanisms (7), mainly inducing the mesenchymal–epithelial transition (8), inhibiting pro-oncogenic pathway in cancer stem cells (30), upregulating miRNAs that reduce oncogene protein expression (8), and downregulating the expression of multi-drug-resistant protein 1 (MRP1) and multi-drug-resistant protein 5 (MRP5), efflux proteins that cause the chemotherapy and multi-kinase drugs to pump out (31, 32). The results of the current study demonstrated for the first time that VitD can resensitize Exo EveR JHH-6 and PLC/PRF/5 to EVE treatment; indeed, VitD when combined with EVE can inhibit cell proliferation and colony forming, overcoming EVE resistance induced by internalization of EveR exosomes. Further studies need to address the underlying mechanisms through which VitD acts in this experimental setting.

In conclusion, the findings of the current study demonstrated that exosomes, released by HCC cell lines induced to be EVE-resistant by drug chronic exposure, may prompt drug resistance in HCC cell lines in terms of cell proliferation and clonal expansion. The exosome-related drug resistance is due at least to the acquired mesenchymal phenotype and to the deregulation of the mTOR pathway through cargo signals. Moreover, the results of the present study proved that VitD may resensitize HCC cells to the exosome-related EVE resistance.

## Data Availability Statement

The raw data supporting the conclusions of this article will be made available by the authors, without undue reservation.

## Author Contributions

MN, FA, AG, RobP, and TM performed experiments, analyzed data, and prepared figures. CdA, CS, and RPir performed literature search, contributed to the interpretation of the data, and provided technical assistance. RA critically revised the manuscript. AC and RPiv provided a significant expert contribution in the scientific content revision process. CP conceived the project, designed the experiments, wrote the manuscript and supervised the manuscript drafting, and critically reviewed and revised it for important intellectual content. All authors contributed to the article and approved the submitted version.

## Conflict of Interest

The authors declare that the research was conducted in the absence of any commercial or financial relationships that could be construed as a potential conflict of interest.

## Publisher’s Note

All claims expressed in this article are solely those of the authors and do not necessarily represent those of their affiliated organizations, or those of the publisher, the editors and the reviewers. Any product that may be evaluated in this article, or claim that may be made by its manufacturer, is not guaranteed or endorsed by the publisher.

## Supplementary Material

Supplementary Figure 1 Schematic protocol summarizing the sequential steps for the isolation of exosomes from HCC resistant cells and for the internalization in HCC parental cells. On day zero, 1.5x10_6_ JHH-6 EveR, 2x10_6_ PLC/PRF/5 EveR, 0.5x10_6_ JHH-6 and 1x10_6_ PLC/PRF/5 parental cells were seeded in 75 cm_2_ flasks in exosome-free medium and grown at 37°C in a humidified atmosphere with 5% CO_2_. After 3 days and every 3 days, precisely at the day 6, 9, 12 and 15, exosomes were isolated from EveR exosome-free medium by Cell Culture Exosome Purification Midi Kit, according to the manufacture instructions and inoculated in the flasks containing parental cells to allow the complete cellular internalization of exosomes. On days 6 and 12, after the collection of media for the exosome isolation, EveR cells, that reached the confluence, were trypsinized and 1.5x10_6_ JHH-6 EveR and 2x10_6_ PLC/PRF/5 EveR were seeded again in a new 75 cm_2_ flasks in exosome-free medium and grown at 37°C in a humidified atmosphere with 5% CO_2_. Alike, on days 8 and 14, parental cells, already exposed to exosomes in the previous days, were trypsinized and 0.5x10_6_ JHH-6 and 1x10_6_ PLC/PRF/5 parental cells were seeded again in a new 75cm2 flasks in exosome-free medium and grown at 37°C in a humidified atmosphere with 5% CO_2_.

## References

[1] Villanueva A. Hepatocellular Carcinoma. N Engl J Med (2019) 380(15):1450–62. doi: 10.1056/NEJMra1713263 30970190

[2] Forner A Reig M Bruix J. Hepatocellular Carcinoma. Lancet (2018) 391(10127):1301–14. doi: 10.1016/S0140-6736(18)30010-2 29307467

[3] Shibue T Weinberg RA. Emt, Cscs, and Drug Resistance: The Mechanistic Link and Clinical Implications. Nat Rev Clin Oncol (2017) 14(10):611–29. doi: 10.1038/nrclinonc.2017.44 PMC5720366 28397828

[4] Aleksakhina SN Kashyap A Imyanitov EN. Mechanisms of Acquired Tumor Drug Resistance. Biochim Biophys Acta Rev Cancer (2019) 1872(2):188310. doi: 10.1016/j.bbcan.2019.188310 31442474

[5] Kalluri R. The Biology and Function of Exosomes in Cancer. J Clin Invest (2016) 126(4):1208–15. doi: 10.1172/JCI81135 PMC4811149 27035812

[6] Qu Z Wu J Wu J Luo D Jiang C Ding Y. Exosomes Derived From Hcc Cells Induce Sorafenib Resistance in Hepatocellular Carcinoma Both *In Vivo* and *In Vitro* . J Exp Clin Cancer Res (2016) 35(1):159. doi: 10.1186/s13046-016-0430-z 27716356 PMC5045585

[7] Negri M Gentile A de Angelis C Monto T Patalano R Colao A. Vitamin D-Induced Molecular Mechanisms to Potentiate Cancer Therapy and to Reverse Drug-Resistance in Cancer Cells. Nutrients (2020) 12(6):1798. doi: 10.3390/nu12061798 PMC7353389 32560347

[8] Provvisiero DP Negri M de Angelis C Di Gennaro G Patalano R Simeoli C. Vitamin D Reverts Resistance to the Mtor Inhibitor Everolimus in Hepatocellular Carcinoma Through the Activation of a Mir-375/Oncogenes Circuit. Sci Rep (2019) 9(1):11695. doi: 10.1038/s41598-019-48081-9 31406139 PMC6690984

[9] Pivonello C Negri M De Martino MC Napolitano M de Angelis C Provvisiero DP. The Dual Targeting of Insulin and Insulin-Like Growth Factor 1 Receptor Enhances the Mtor Inhibitor-Mediated Antitumor Efficacy in Hepatocellular Carcinoma. Oncotarget (2016) 7(9):9718–31. doi: 10.18632/oncotarget.6836 PMC4891079 26756219

[10] Pivonello C Rousaki P Negri M Sarnataro M Napolitano M Marino FZ. Effects of the Single and Combined Treatment With Dopamine Agonist, Somatostatin Analog and Mtor Inhibitors in a Human Lung Carcinoid Cell Line: An *In Vitro* Study. Endocrine (2017) 56(3):603–20. doi: 10.1007/s12020-016-1079-2 27688013

[11] Hofland LJ van Koetsveld PM Lamberts SW. Percoll Density Gradient Centrifugation of Rat Pituitary Tumor Cells: A Study of Functional Heterogeneity Within and Between Tumors With Respect to Growth Rates, Prolactin Production and Responsiveness to the Somatostatin Analog Sms 201-995. Eur J Cancer (1990) 26(1):37–44. doi: 10.1016/0277-5379(90)90254-q 2138476

[12] Franken NA Rodermond HM Stap J Haveman J van Bree C. Clonogenic Assay of Cells *In Vitro* . Nat Protoc (2006) 1(5):2315–9. doi: 10.1038/nprot.2006.339 17406473

[13] Zhao H Desai V Wang J Epstein DM Miglarese M Buck E. Epithelial-Mesenchymal Transition Predicts Sensitivity to the Dual Igf-1r/Ir Inhibitor Osi-906 in Hepatocellular Carcinoma Cell Lines. Mol Cancer Ther (2012) 11(2):503–13. doi: 10.1158/1535-7163.MCT-11-0327 22161861

[14] Serova M Tijeras-Raballand A Dos Santos C Albuquerque M Paradis V Neuzillet C. Effects of Tgf-Beta Signalling Inhibition With Galunisertib (Ly2157299) in Hepatocellular Carcinoma Models and in *Ex Vivo* Whole Tumor Tissue Samples From Patients. Oncotarget (2015) 6(25):21614–27. doi: 10.18632/oncotarget.4308 PMC4673290 26057634

[15] Klemm F Joyce JA. Microenvironmental Regulation of Therapeutic Response in Cancer. Trends Cell Biol (2015) 25(4):198–213. doi: 10.1016/j.tcb.2014.11.006 25540894 PMC5424264

[16] Li S Yi M Dong B Jiao Y Luo S Wu K. The Roles of Exosomes in Cancer Drug Resistance and Its Therapeutic Application. Clin Transl Med (2020) 10(8):e257. doi: 10.1002/ctm2.257 33377643 PMC7752167

[17] Zhang X Yuan X Shi H Wu L Qian H Xu W. Exosomes in Cancer: Small Particle, Big Player. J Hematol Oncol (2015) 8:83. doi: 10.1186/s13045-015-0181-x 26156517 PMC4496882

[18] Li I Nabet BY. Exosomes in the Tumor Microenvironment as Mediators of Cancer Therapy Resistance. Mol Cancer (2019) 18(1):32. doi: 10.1186/s12943-019-0975-5 30823926 PMC6397467

[19] He M Qin H Poon TC Sze SC Ding X Co NN. Hepatocellular Carcinoma-Derived Exosomes Promote Motility of Immortalized Hepatocyte Through Transfer of Oncogenic Proteins and Rnas. Carcinogenesis (2015) 36(9):1008–18. doi: 10.1093/carcin/bgv081 26054723

[20] Obenauf AC Zou Y Ji AL Vanharanta S Shu W Shi H. Therapy-Induced Tumour Secretomes Promote Resistance and Tumour Progression. Nature (2015) 520(7547):368–72. doi: 10.1038/nature14336 PMC4507807 25807485

[21] Chen L Guo P He Y Chen Z Chen L Luo Y. Hcc-Derived Exosomes Elicit Hcc Progression and Recurrence by Epithelial-Mesenchymal Transition Through Mapk/Erk Signalling Pathway. Cell Death Dis (2018) 9(5):513. doi: 10.1038/s41419-018-0534-9 29725020 PMC5938707

[22] Qu Z Feng J Pan H Jiang Y Duan Y Fa Z. Exosomes Derived From Hcc Cells With Different Invasion Characteristics Mediated Emt Through Tgf-Beta/Smad Signaling Pathway. Onco Targets Ther (2019) 12:6897–905. doi: 10.2147/OTT.S209413 PMC6711569 31692540

[23] Sciammarella C Luce A Riccardi F Mocerino C Modica R Berretta M. Lanreotide Induces Cytokine Modulation in Intestinal Neuroendocrine Tumors and Overcomes Resistance to Everolimus. Front Oncol (2020) 10:1047. doi: 10.3389/fonc.2020.01047 32766136 PMC7379869

[24] Dowling RJ Topisirovic I Alain T Bidinosti M Fonseca BD Petroulakis E. Mtorc1-Mediated Cell Proliferation, But Not Cell Growth, Controlled by the 4e-Bps. Science (2010) 328(5982):1172–6. doi: 10.1126/science.1187532 PMC2893390 20508131

[25] Nishi T Iwasaki K Ohashi N Tanaka C Kobayashi D Nakayama G. Phosphorylation of 4e-Bp1 Predicts Sensitivity to Everolimus in Gastric Cancer Cells. Cancer Lett (2013) 331(2):220–9. doi: 10.1016/j.canlet.2013.01.004 23340172

[26] Zhou Q Wong CH Lau CP Hui CW Lui VW Chan SL. Enhanced Antitumor Activity With Combining Effect of Mtor Inhibition and Microtubule Stabilization in Hepatocellular Carcinoma. Int J Hepatol (2013) 2013:103830. doi: 10.1155/2013/103830 23509629 PMC3590758

[27] Tabernero J Rojo F Calvo E Burris H Judson I Hazell K. Dose- and Schedule-Dependent Inhibition of the Mammalian Target of Rapamycin Pathway With Everolimus: A Phase I Tumor Pharmacodynamic Study in Patients With Advanced Solid Tumors. J Clin Oncol (2008) 26(10):1603–10. doi: 10.1200/JCO.2007.14.5482 18332469

[28] Tamburini J Chapuis N Bardet V Park S Sujobert P Willems L. Mammalian Target of Rapamycin (Mtor) Inhibition Activates Phosphatidylinositol 3-Kinase/Akt by Up-Regulating Insulin-Like Growth Factor-1 Receptor Signaling in Acute Myeloid Leukemia: Rationale for Therapeutic Inhibition of Both Pathways. Blood (2008) 111(1):379–82. doi: 10.1182/blood-2007-03-080796 17878402

[29] Vilar E Perez-Garcia J Tabernero J. Pushing the Envelope in the Mtor Pathway: The Second Generation of Inhibitors. Mol Cancer Ther (2011) 10(3):395–403. doi: 10.1158/1535-7163.MCT-10-0905 21216931 PMC3413411

[30] Zheng W Duan B Zhang Q Ouyang L Peng W Qian F. Vitamin D-Induced Vitamin D Receptor Expression Induces Tamoxifen Sensitivity in Mcf-7 Stem Cells *Via* Suppression of Wnt/Beta-Catenin Signaling. Biosci Rep (2018) 38(6):BSR20180595. doi: 10.1042/BSR20180595 30314996 PMC6435469

[31] Tan KW Sampson A Osa-Andrews B Iram SH. Calcitriol and Calcipotriol Modulate Transport Activity of Abc Transporters and Exhibit Selective Cytotoxicity in Mrp1-Overexpressing Cells. Drug Metab Dispos (2018) 46(12):1856–66. doi: 10.1124/dmd.118.081612 PMC7333660 30232176

[32] Gilzad-Kohan H Sani S Boroujerdi M. Calcitriol Reverses Induced Expression of Efflux Proteins and Potentiates Cytotoxic Activity of Gemcitabine in Capan-2 Pancreatic Cancer Cells. J Pharm Pharm Sci (2017) 20(0):295–304. doi: 10.18433/J37W7R 28885916

