# Supplementary figures and images for: Vitamin D Reverts the Exosome-Mediated Transfer of Cancer Resistance to the mTOR Inhibitor Everolimus in Hepatocellular Carcinoma
Source: Front Oncol. 2022 Apr 25;12:874091. doi: 10.3389/fonc.2022.874091 (PMC9083073; doi:10.3389/fonc.2022.874091)

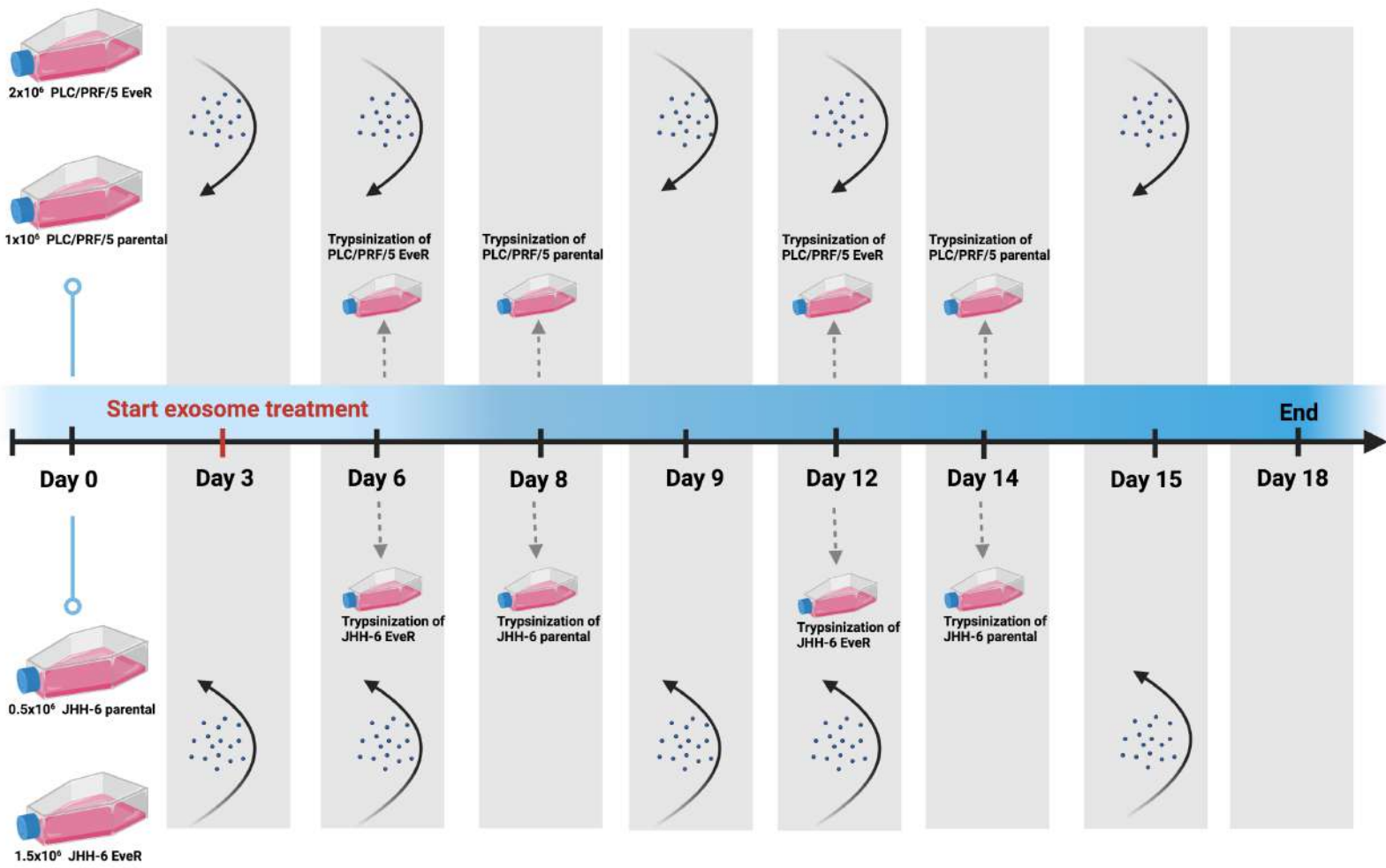

Supplement: Supplementary Figure 1 — Schematic protocol summarizing the sequential steps for the isolation of exosomes from HCC resistant cells and for the internalization in HCC parental cells. On day zero, 1.5x10^6^ JHH-6 EveR, 2x10^6^ PLC/PRF/5 EveR, 0.5x10^6^ JHH-6 and 1x10^6^ PLC/PRF/5 parental cells were seeded in 75 cm2 flasks in exosome-free medium and grown at 37°C in a humidified atmosphere with 5% CO2. After 3 days and every 3 days, precisely at the day 6, 9, 12 and 15, exosomes were isolated from EveR exosome-free medium by Cell Culture Exosome Purification Midi Kit, according to the manufacture instructions and inoculated in the flasks containing parental cells to allow the complete cellular internalization of exosomes. On days 6 and 12, after the collection of media for the exosome isolation, EveR cells, that reached the confluence, were trypsinized and 1.5x10^6^ JHH-6 EveR and 2x10^6^ PLC/PRF/5 EveR were seeded again in a new 75 cm2 flasks in exosome-free medium and grown at 37°C in a humidified atmosphere with 5% CO2. Alike, on days 8 and 14, parental cells, already exposed to exosomes in the previous days, were trypsinized and 0.5x10^6^ JHH-6 and 1x10^6^ PLC/PRF/5 parental cells were seeded again in a new 75cm2 flasks in exosome-free medium and grown at 37°C in a humidified atmosphere with 5% CO2. [file Image_1.pdf]
